# Design and Usability of a Digitalized Compensatory Goal Management Training for Individuals with Brain Injury: A User-Centered Approach

**DOI:** 10.63144/ijt.2025.6718

**Published:** 2025-12-12

**Authors:** Elise M. Verhoog, Roy P.C. Kessels, Luciano Fasotti, Dirk Bertens

**Affiliations:** 1 Radboud University, Donders Institute for Brain, Cognition and Behaviour, Nijmegen, The Netherlands; 2 Rehabilitation Centre Klimmendaal, Arnhem, The Netherlands; 3 Vincent van Gogh Institute for Psychiatry, Venray, The Netherlands

**Keywords:** Brain injury, Executive function, Goal Management Training, Neuropsychological rehabilitation, Serious games

## Abstract

Although there is more evidence in neuropsychological rehabilitation for transfer of treatment effects to daily life when using compensatory strategy training, computerized programs for patients with acquired brain injury still focus on function training. Therefore, we developed Karman Line Plan, a digitalized version of an evidence-based compensatory Goal Management Training intervention for individuals with brain injury and executive function problems. Karman Line Plan consists of a digital environment (Plan Game) and a mobile application (Plan Tool). This study aims to describe the design process and evaluate the usability and acceptance of Karman Line Plan. The program was developed using a three-phase standardized user-centred design approach. Results indicate that Karman Line Plan is perceived as user-friendly and valuable by both patients and therapists. The findings emphasize the importance of involving the target patient population early in the development process to allow for early adjustments and meet end-user needs.

Individuals with brain injuries in the chronic phase commonly encounter significant cognitive challenges, such as problems with attention, memory, speed of information processing and/or executive function (i.e. the ability to plan and execute goal-directed behaviour), affecting their functioning in daily life. The objective of neuropsychological rehabilitation is to help these individuals in achieving their highest potential and maximal independence, thereby facilitating their potential return to their homes, autonomous performance of activities, and integration into society (Wilson, 2008). To achieve this goal, a broad range of training programs is provided to individuals with brain injuries. Such neuropsychological rehabilitation programs typically span several months, are costly, and require ongoing commitment and active involvement from the affected individuals (Bayley et al., 2023; Cicerone et al., 2019). Consequently, such training programs may sometimes cause stress, frustration, and participant attrition due to the comprehensive nature of the interventions (Tuah et al., 2021). A shift from traditional in-person neuropsychological rehabilitation interventions to digitalised, playful, and interactive programs could reduce the patient burden, as well as the health-care cost, and may lead to more favourable outcomes in the rehabilitation process of cognitive impairments, as well as in behavioural changes. The use of serious games and gamification in neuropsychological rehabilitation may be a motivational driver to enhance individual engagement during the rehabilitation process and has high potential for at-home personalised use, suggesting real promise in the field of neuropsychological rehabilitation (Khaleghi et al., 2021; Sigmundsdottir et al., 2016; Tuah et al., 2021). Over the past decade, there has been a concomitant increase in both the number of Gamified Cognitive Training (GCT) programs and of scientific studies exploring the benefits of this type of intervention.

Historically, neuropsychological rehabilitation has been based on two theoretically distinct approaches, that is, (1) the restoration of compromised cognitive functions through (computerized) function training, or (2) compensating for cognitive dysfunctions through cognitive strategy training (Johnson & Weinberg, 2023; Wilson et al., 2017). In the last two decades, the restorative approach has seen a tremendous growth in GCT programs often referred to as “brain training” or “brain games.” These GCT programs are based on the rationale that by performing mental exercises of increasing difficulty, impaired cognitive functions that underpin real-world functioning can be trained (i.e., restored) (Simons et al., 2016; Green & Bavelier, 2008). Although some restorative based GCT programs, such as Cogmed®, Lumosity^TM^ or Rehacom®, were initially considered promising based on the then-available evidence (Björkdahl et al., 2013; Lundqvist et al., 2010; Mam Khezri & Mikaeli Manieh, 2021; Veisi-Pirkoohi et al., 2020; Westerberg et al., 2007), independent research of such gamified types of function training has repeatedly questioned their effectiveness – and sometimes bold claims (Sigmundsdottir et al., 2016; Van Heugten et al., 2016). Positive efficacy studies are in most cases based on cognitive outcome measures, which often highly resemble the tasks practiced within the GCT programs, rather than on assessments of daily life functioning. As a result, evidence for successful transfer to everyday life of these restorative based GCT treatments is scarce (Sigmundsdottir et al., 2016).

Evidence of interventions focused on “compensation” rather than on “restoration” of cognitive functions is more promising (Bayley et al., 2023; Cicerone et al., 2019). The compensatory approach focuses on learning skills and strategies to overcome cognitive problems and relearn demanding daily life activities (Wilson et al., 2017). Accordingly, the use of different types of strategy training for daily-life demands that rely on specific cognitive domains is currently recommended as a practice standard (Cicerone et al., 2011), since these types of interventions currently have the best level of evidence when it comes to transfer of treatment effects to daily life (Bayley et al., 2023; Cicerone et al., 2019).

One of the most widely used and extensively studied cognitive strategy training programs is Goal Management Training (GMT) (Levine et al., 2000; Robertson, 1996). GMT is an evidence-based intervention widely used in neuropsychological rehabilitation practice, aimed at improving executive problems in individuals with acquired brain injury. Executive functions, including higher-order cognitive processes such as planning, organizing, and goal-directed behaviour, are one of the core cognitive domains affected in many patients with ABI (Stuss, 2011). Problems with the executive functions result in disorganized behaviour and increased distractibility during the performance of everyday tasks. As a result, GMT is aimed at teaching individuals to compensate for deficits in these processes by providing them with an algorithm consisting of five stages (Levine et al., 2000; Robertson, 1996). As can be seen in Figure 1, these stages are: stage 1: Stop and direct your attention towards the task; stage 2: Define the main goal; stage 3: Divide the goal into subgoals or steps; stage 4: Rehearse the steps; stage 5: Perform them and monitor the performance by comparing the outcome of action with the description of the subgoal/step (Van Hooren et al., 2007). Individuals with brain injuries thus learn to apply these instructions during the performance of complex instrumental activities of daily living such as making a shopping list, cleaning the house, or doing the financial administration, all of which contribute to functional independence. GMT has been shown to be an effective intervention in which intervention effects generalize to untrained instrumental activities of daily living (IADL) (i.e., generalization) (Stamenova & Levine, 2018).

While GMT as a treatment has been found to be effective, it is inherently a lengthy intervention that spans several months and necessitates participants to engage in repetitive task exercises and strategy rehearsals. As an extension to the in-person “compensation-based” GMT, we have therefore developed a strategic game that can be played on a tablet, laptop or personal computer (PC) and a mobile application for smartphone use. This game may potentially reduce the amount of in-person guidance with the therapist and facilitates independently in-home practice of the GMT strategy using a safe digital environment. The strategic game differs from existing brain games in that it specifically focuses on teaching a compensatory strategy rather than on the repetitive stimulation of a specific cognitive function with the aim of restoration. Designing a gamified GMT with its compensatory aim requires more effort than creating games with the purpose of maximizing cognitive effort through intensive and repeated practice. In strategic games, consideration should be given to the following three factors. First, patients need to become more aware of their own cognitive problems and need to recognise when difficulties are likely to occur in specific situations during the game. Also, novel strategies need to be introduced and practiced in varying tasks and situations. Finally, patients not only need to learn how and when the strategy should be applied in the game, but also in real-life situations (Geusgens et al., 2007; Kuil et al., 2018). In summary, the requirements for implementing a “compensationbased” GCT program in clinical populations highlights the need for further investigation into its applicability and feasibility.

## Objectives

The current project encompasses the development and assessment of the feasibility of a “compensation-based” gamified version of GMT for the treatment of executive problems in individuals with brain injuries in the chronic phase. More specifically, the focus of this study is both (1) to describe and discuss the design process of the gamified version of GMT based on user-requirements, advice of patients and therapists, and iterative testing; and (2) to examine its usability and user acceptance after the compensation-based gamified version of GMT has been implemented as a treatment for the rehabilitation of executive function problems. The results of this work will contribute to the improvement of user-centered tailoring of gamification in cognitive training programs and will provide valuable insights into its applicability and potential benefits in clinical practice.

## Method

### Overview

As an extension to the evidence-based in-person compensatory Goal Management Training (GMT), a game and a mobile application (referred to as Karman Line Plan) were developed. Karman Line Plan is part of the Karman Line game series, one of the first set of games based on evidence-based compensation strategies used in neuropsychological rehabilitation for the treatment of several cognitive problems such as information processing speed (Karman Line Tempo), memory (Karman Line Memory) and fatigue (Karman Line Energy)(www.karmanlinedtx.com). The “Karman Line Plan module” combines in-person GMT with a game that can be played on a tablet, laptop or PC (i.e., Plan Game, see Figure 4 and 5) and an additional mobile application developed for smartphone use (i.e., Plan Tool, see Figure 3). While playing the Plan Game, patients learn to apply the GMT strategy to various digitally simulated tasks that require executive functions, such as planning a route or preparing a meal. The Plan Tool supports the participant in regaining control during the performance tasks. Both the Plan Game and Plan Tool were developed and optimized by adopting a user-centered approach (Procci et al., 2012), based on three phases spread over a three-year period (March 2020 – April 2023), to guarantee that potentially important aspects, such as usability and user-acceptance, were not overlooked at any phase of the development process (see Figure 2). In order to provide a clear overview on how the user-centered feedback from each phase was used in the subsequent phases of the development process, we have described the methodology and corresponding results per phase, i.e., phase 1: pre-development, phase 2: prototype development, and phase 3: implementation in rehabilitation practice.

### Phase 1: Pre-development

#### Methods and Participants

The aim of the first phase was to properly identify the target audience and user characteristics. During the initial gathering of user characteristics, multidisciplinary sessions were held for one hour once a week over the course of 7 months. The multidisciplinary pre-development sessions were held with different disciplines, including three clinical neuropsychologists, two neuropsychological researchers, two front-end game designers, one back-end designer and programmer, and an artificial intelligence expert. The main aims of the multidisciplinary (brainstorm) sessions were (1) to establish the requirements of the game, that is, duration, number of game levels, design, 2D or 3D orientation, the game’s character’s first-person perspective (i.e., the player sees the world from the character’s viewpoint) or isometric perspective (i.e., the player sees the world from a fixed point in a three-dimensional environment), and type of instructions (verbal or visual); and (2) to find out characteristics of the end user that may influence the interaction with the game (i.e., reading level, gaming experience, and other capabilities and limitations due to brain injury such as executive disabilities, sensory overload, and limitations in information processing that may all impact the interaction with the game). In addition, we arranged meetings with individuals with acquired brain injury as potential end users (*N* = 3) and interviewed them to ensure that potentially important details were not overlooked, and to determine the demands of the task and designs. We showed them different designs and asked their preferences about different game elements, such as: How do you feel about the (1) gameplay, (2) style, (3) colour tones, (4) amount of information load per screen, and (5) are the characters a good fit (see Table 1 for statements that are used to evaluate the likelihood of the design). We also determined whether the end-user experienced a need or desire to use a game for learning the GMT strategy, or whether they were hesitant or experienced resistance. Once the requirements and user characteristics were generated and collected, a storyboard and paper prototype were developed in the next phase.

### Phase 2: Development Prototype

**Figure 1:**
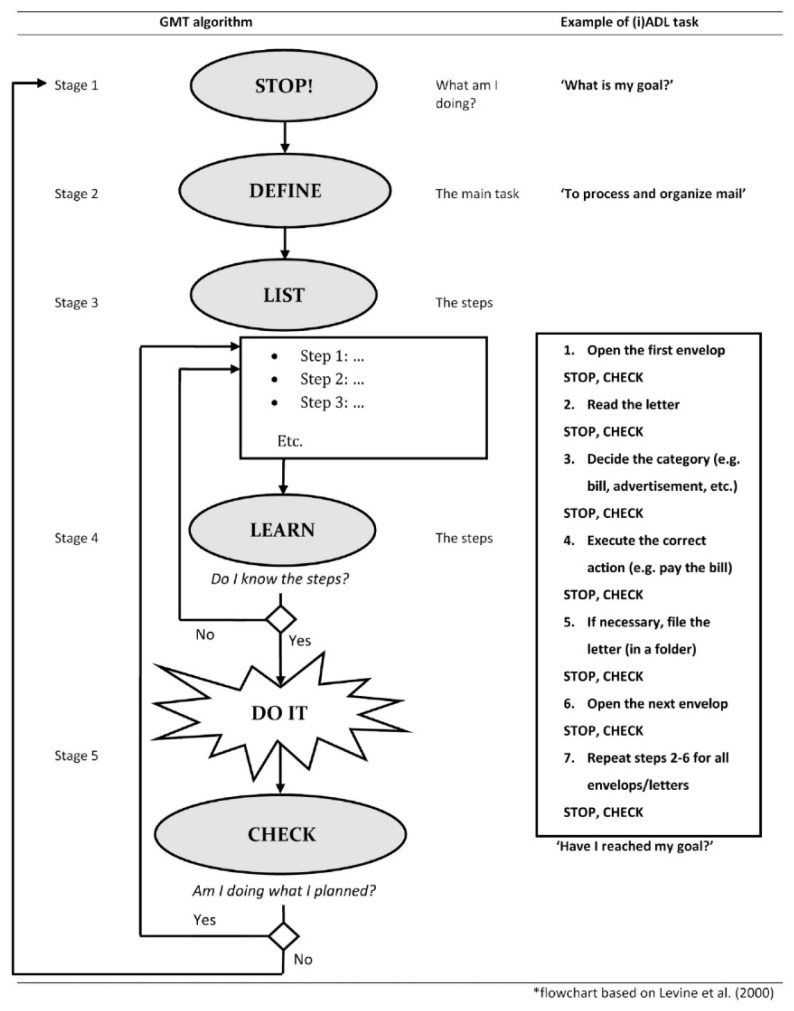
Flowchart of the GMT Algorithm and an Illustrative Example of Its Application *Note*. Derived from Bertens et al. (2013)

**Figure 2:**
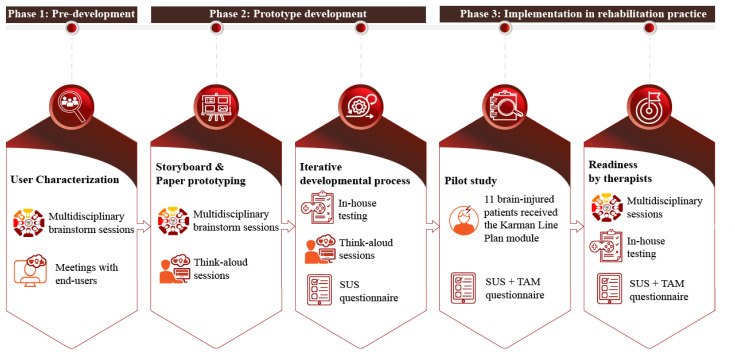
Project Schedule

The purpose of the second phase (see Figure 1) was to design and develop a prototype of the Karman Line Plan module (phase 2.1) by using an iterative, incremental developmental process (phase 2.2).

#### Phase 2.1: Storyboard and Paper Prototyping

##### Methods and participants

The first step in developing the prototype was to generate the concepts for the game in storyboard format, including game play, style, colour tones, and design while taking the end user and multidisciplinary considerations from Phase 1 into account. Subsequently, paper prototypes of the Plan Game and Plan Tool were developed based on repeated multidisciplinary sessions of one hour once a week over the course of 14 months. The paper prototype served as a representation of the Plan Game and Plan Tool displaying the structure and elements of the screen flow, allowing researchers to show the game concept to the end users (i.e., patients with brain injuries) through think-aloud sessions via virtual meetings.

During the think-aloud sessions, end users were asked to say whatever came into their mind when we showed them the hand-sketched “screens” of the (digital) Plan Game and Plan Tool. The researcher took notes and gathered information and opinions about the Plan Game and Plan Tool, with regard to its structure, characters, storyline, readability, flow, and overall presentation. The end users in this phase consisted of a group of young (*N* = 10, aged: 23 – 32 years) and middle-aged (*N* = 12, aged: 44 – 62 years) individuals with brain injury in the chronic phase (> 3 months post-injury). We aimed to harness the middle-aged individuals with brain injury opinions alongside the views of young individuals with brain injury, who are hypothesized to embrace technology more readily.

The recruitment process was conducted differently for the young and middle-aged individuals with brain injury. We collaborated with the Van der Sar Foundation (i.e., a Dutch foundation that develops projects to support people with brain injury; https://www.hersenstichting.nl/onderzoeken-en-projecten/projecten-edwin-van-der-sar-foundation-archief/) for the recruitment of the young individuals. Social media-based recruitment in peer support groups was conducted for the middle-aged individuals with brain injury. Table 2 shows the demographic information of the participants.

Feedback was collected using multiple sessions with smaller samples from the participants. More specifically, following the think-aloud session with a random sample from the young-aged group (*N* = 3) on one level of the Plan Game, the developers worked to integrate the feedback from the young end-users and moved the game level forward for the next think aloud session with a random sample from the middle-aged end-users (*N* = 3).

The think-aloud sessions with different young and middle-aged end-users were repeated for all the levels of the Plan Game that consisted of a different gameplay and flow. In addition, the paper prototype of the Plan Tool was reviewed by using think-aloud sessions with three young and three middle-aged participants (*N* = 6), randomly selected from the sample. The outcome from this repeated and iterative process was an end-user accepted paper prototype for all levels of the Plan Game and the Plan Tool which could be translated into a rough first digital prototype.

#### Phase 2.2: Iterative Developmental Process

##### Methods and participants

Once we had updated the paper prototypes of all Plan Game levels and the Plan Tool with feedback from the think-aloud sessions, the programmers created a digital prototype of the first four levels of the Plan Game. The usability testing proceeded in the same manner as in Phase 2.1, taking 11 months to complete. Upon completion of the first digital build, in-house testing took place involving researchers and game designers. The in-house test sessions involved testing the functionality and limitations of the game to identify the most glaring usability problems so that future iterations could focus on the more concise, population specific problems. Errors and bugs were logged in a bug tracker (Microsoft Azure DevOps), and any other issues, such as unclear text, were recorded in a report. All the suggested changes were discussed in the multidisciplinary team and were implemented where appropriate.

After the suggested changes from the in-house test sessions were integrated, a small usability study, with three young and three middle-aged individuals with brain injury (*N* = 6) was completed through think-aloud sessions to ensure that any population-specific usability issues were addressed. Individuals with brain injury were instructed to express their thoughts when they experienced difficulties during gameplay, when they needed to ask for instruction, or when they encountered something worth commenting upon. The end-users were also encouraged to let the researcher know when they felt confused, lost, or frustrated. Explicit problems and specific design suggestions were listed in the report. This process was iterative and was repeated for the development of all Plan Game levels.

The iterative development process for the smartphone application was dealt with in the same manner as for the Plan Game. All participating end-users with brain injuries were also asked to rate the usability of the Plan Tool on the System Usability Scale (SUS) questionnaire (Lewis, 2018). The SUS questionnaire consists of ten items scored on a 5-point Likert scale (1=strongly disagree, 5=strongly agree) with a maximum score of 100. The sum scores on the SUS questionnaire can be interpreted as worst imaginable (12.5 – 20.2), awful (20.3 – 35.6), poor (35.7 – 50.8), OK (50.9 – 71.3), good (71.4 – 85.4), excellent (85.5 – 90.8), or best imaginable (90.9 – 100). The psychometric properties of the scale have been positively evaluated (Lewis, 2018). The quantitative metrics helped to specifically determine what the end-users liked about the Plan Tool, and what needed to be changed before they would consider using it in their daily life.

**Figure 3:**
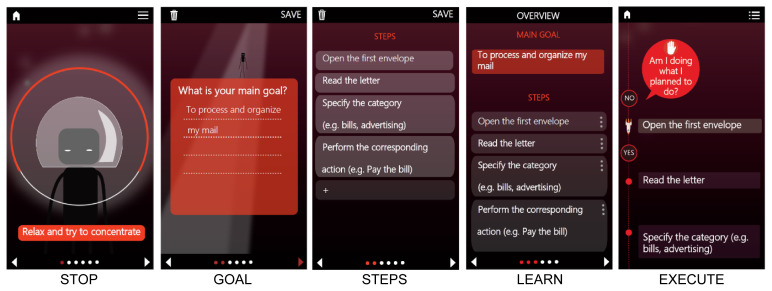
Screenshots of the Mobile Application for Smartphone Use, Named Plan Tool

**Figure 4:**
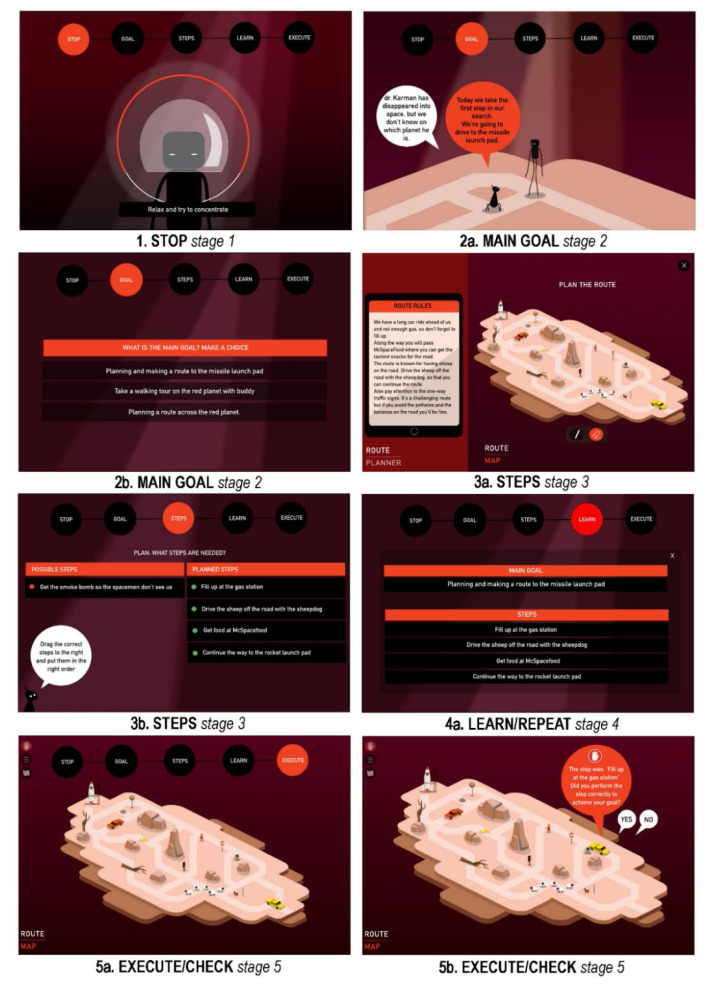
GMT Strategy Components Within a Single Game Level

As a final check, we tested the adapted Plan Game levels and Plan Tool by adopting think-aloud sessions with four cognitive therapists working at Rehabilitation Center Klimmendaal, and one individual with brain injury (*N* = 1) who had also received the conventional GMT treatment at the outpatient clinic of Rehabilitation Center Klimmendaal. The outcome from this repeated and iterative process was an end user accepted digital prototype containing the Plan Tool and 16 levels of the Plan Game (see Figures 3 and 4).

In the Plan Tool, specific everyday activities can be added and executed following the different GMT stages. As can be seen in Figure 3, the strategy is applied in the app for an individual who experiences difficulties with processing and organizing mail. An individual starts with stage 1 of the strategy: STOP, try to concentrate yourself and direct your attention towards the task; next, the individuals enter stage 2 in which they have to specify their main GOAL, e.g., ‘to process and organize my mail.’ In stage 3, they then must divide the task into subgoals or STEPS. In stage 4, they have to LEARN/rehearse their goal and the steps before starting to perform the task in stage 5. During execution, people are then guided step-by-step through the task execution, each time being asked to check their performance by comparing the outcome of their action with the description of the subgoal/step.

### Phase 3: Implementation in Rehabilitation Practice

#### Phase 3.1: Pilot Study

##### Methods and participants

This phase focused on the evaluation of the usability and target-user acceptance of the Karman Line Plan module. All feedback from phase 2 was implemented, resulting in an optimized digital prototype containing the Plan Tool and 16 levels of the Plan Game (See Figures 3 and 4). This prototype was subsequently used in a pilot evaluation in which a total of 11 individuals with brain injury received the Karman Line Plan module in combination with a shortened in-person GMT intervention which involved seven sessions with a therapist (see Figure 6). The pilot evaluation is part of a randomized controlled study in which the efficacy of the Karman Line Plan module is evaluated in addition to its usability and user-acceptance. However, the results regarding the efficacy are beyond the scope of this paper and will be reported elsewhere.

For the current pilot evaluation, patients were recruited from the outpatient clinic of Rehabilitation Centre Klimmendaal. Patients were considered eligible if they had any type of acquired brain injury with a post-onset time of more than 3 months, lived independently at home, were aged between 18 and 70 years, and reported subjective executive complaints evidenced by a clinically meaningful (increased; T-score > 65) score on the Behaviour Rating Inventory of Executive Function-Adult Version (BRIEF-A) (Scholte & Noens, 2011). Patients were excluded if they had a neurodegenerative disorder, aphasia, neglect, or were in treatment for severe psychiatric disorders, or could not operate a computer or smartphone. The researcher contacted the patients by phone to provide information about the study and to inquire if they wanted to participate in the study. After a one-week decision period, the informed consent form was signed, and patients started with the Karman Line Plan module.

After playing the Plan Game levels, two questionnaires were employed to inquire the patients’ usability and acceptance opinion about the game prototypes as a training in addition to the in-person therapy sessions. The same holds for the Plan Tool. First, the SUS questionnaire was administered to measure the usability. Second, items from the Technology Acceptance Model (TAM) (Holden & Karsh, 2010) were used to measure the acceptance based on constructs of perceived ease of use, perceived usefulness, perceived enjoyment, and intention to use. Furthermore, semi-structured interviews with patients were carried out to check the findings from the surveys and to gather their experience of using the Plan Game and Plan Tool, and factors that influenced their intention to use the game series. Table 2 outlines the characteristics of the participating end users in the study.

**Figure 5:**
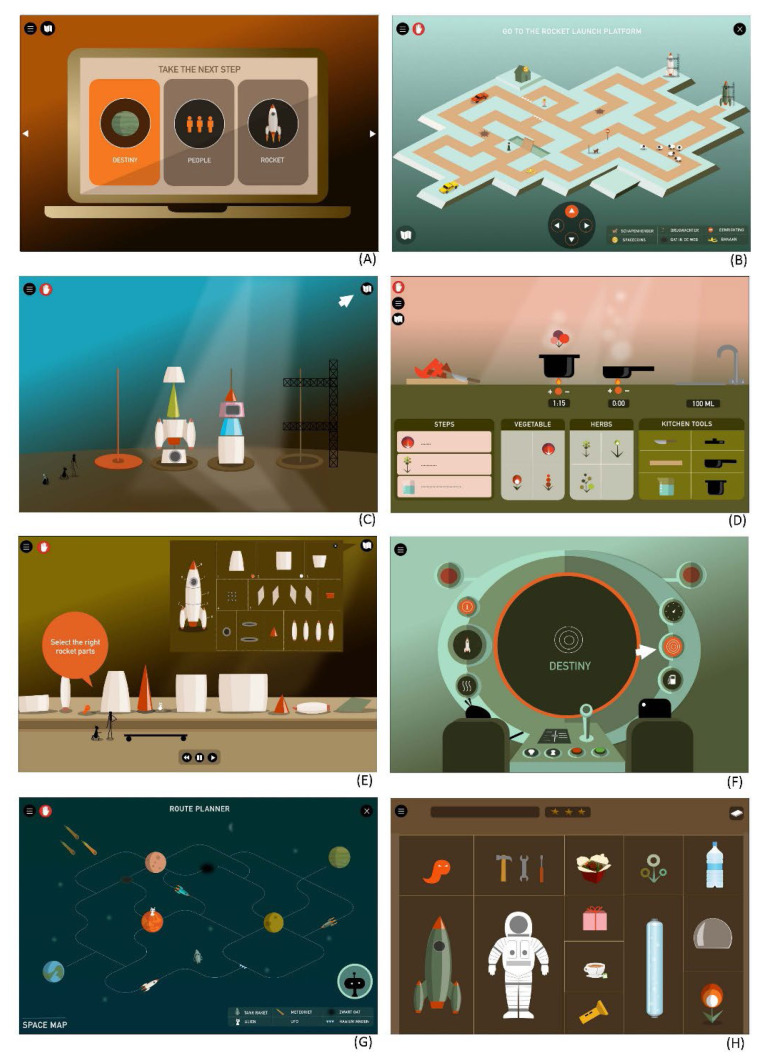
Plan Game Levels

**Figure 6:**
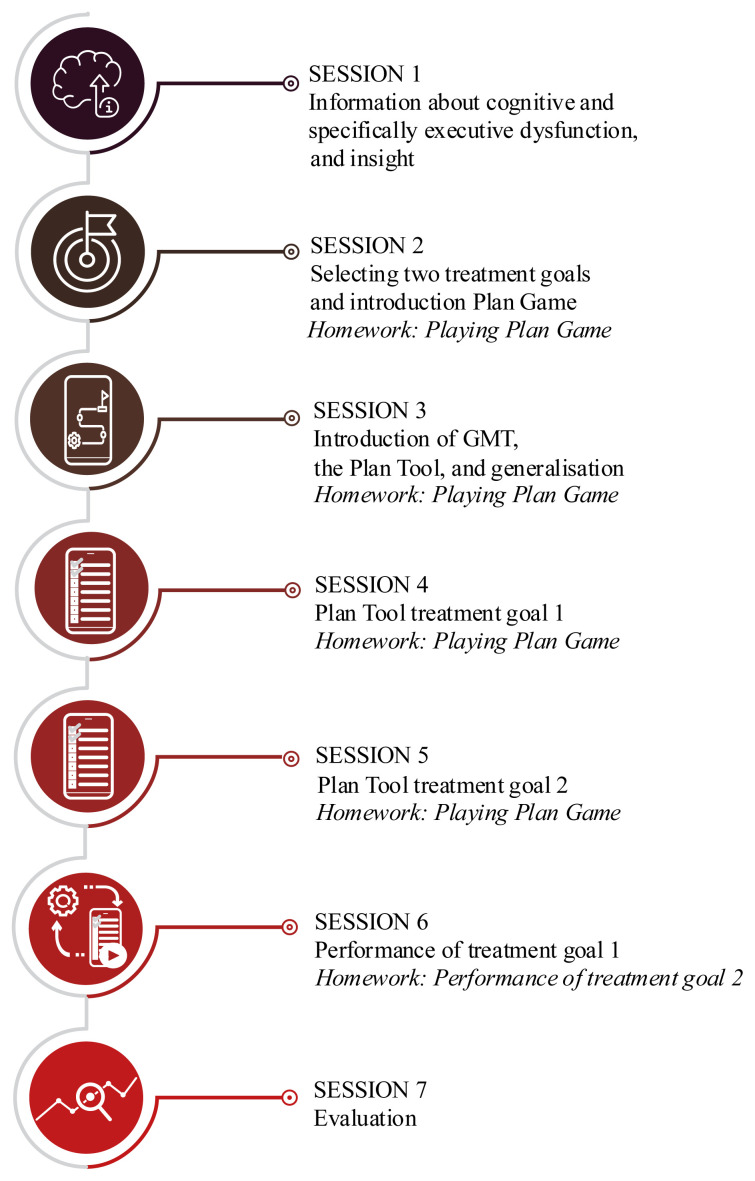
Overview of the Shortened In-person GMT Intervention *Note*. Used in combination with the digitalised Plan module

Figure 4 depicts the various screens that a player encounters while playing a single game level. All stages of the GMT strategy taught to the player are addressed. The game begins with a STOP moment (*Stage 1 STOP*), after which the day’s goal is explained, and the player is challenged to identify the correct goal of the task (*Stage 2 DEFINE*). Then the task steps are described, of which the player must choose the correct steps from a multitude of irrelevant information or construct his or her own steps, and then create a step-by-step plan (*Stage 3 LIST*). Players are then encouraged to thoroughly read and repeat the step-by-step plan (*Stage 4 LEARN*) before putting it into action. Once the player begins executing, they learn to adhere to the step-by-step plan and monitor one’s own behaviour through self-monitoring, ensuring that each action is still in accordance with the plan (*Stage 5 CHECK*). In each game level, the sequence of displays a player must go through is the same. So, the entire GMT strategy is played out throughout every game level. However, this sequence is practised within a wide variety of endeavours (see Figure 5).

Figure 5 depicts various screens from several levels of the Plan Game. It demonstrates how participants practise the application of the strategy in various tasks and situations, such as when booking a flight (A), planning a route (B), building a rocket (C), cooking (D), collecting rocket parts (E), learning how to pilot a rocket (F), traversing a route through the planet (G), and packing for space travel (H).

#### Phase 3.2: Readiness by Therapists

##### Methods and participants

Final issues that were addressed during the pilot evaluation study were addressed, and a final build of the Plan Game and Plan Tool were prepared by adopting input from multidisciplinary brainstorm sessions with the same disciplinary team for one hour once a week, for 3 months. We then conducted one final round of usability testing that contained in-house testing using our own development team and administering the SUS and TAM questionnaires to occupational therapists, cognitive trainers, psychologists and rehabilitation physicians from rehabilitation centre Klimmendaal (*N* = 11). The therapists were asked to meticulously walk through the final version of the Plan Game and Plan Tool before rating the usability on the SUS and TAM questionnaires. This usability round was conducted to ensure the absence of functional bugs in the final versions of the game and app, to fulfil all initial development objectives, and to identify and address potential concerns of therapists, thereby promoting implementation in the clinical practice.

## Results

Within this section, the results from each phase will be presented.

### Pre-development (Phase 1)

Phase 1 focused on the specific requirements and user characteristics as demanded by the multidisciplinary team and persons with brain injury as end users. This information provided a backdrop for the researchers and developers for designing the Plan Game and Plan Tool. More specifically, the findings in this stage indicated that brain-injured end-users preferred a game that could be played on a PC, laptop, or tablet (i.e., Plan Game) and, additionally, a mobile application on their smartphone (i.e., Plan Tool) that could serve as a guide when performing demanding daily activities.

While the Plan Game had to emphasize the practice of strategies through fixed and predetermined tasks and exercises, the Plan Tool had to provide users with the opportunity to incorporate their own problematic daily activities, such as doing the groceries or planning a trip by public transport. Consequently, the Plan Tool had to serve as a direct link to daily life. With regard to the Plan Game, individuals with brain injury indicated that they preferred a 2D flat orientation, an isometric perspective so that they had an overview of the situation they were going to encounter in the game, a point and click (adventure) game, soft colour tones, and visual as well as verbal instructions.

Additionally, the multidisciplinary team debated that the Plan Game and Plan Tool had to serve as a support during (and after) the in-person GMT therapy and not as a substitute for the role of the therapist or the treatment itself. Previous research has shown that therapist-assisted digitalized training interventions were found to be more effective than GCT programs alone (Sigmundsdottir et al., 2016). Also, it was decided that the game levels should be playable directly on the web using WebGL, without the need to download and install any programmes, to ensure applicability in different settings without difficulties. As to the design of the game levels, it was decided that they should be synchronised with the therapist given treatment sessions, to ensure the participants’ weekly practice and application of strategies in different tasks/scenarios. Also, the level of difficulty should gradually increase as treatment progresses, and each game level should consist of a separate task with a clear beginning and end. Performing one game, that is, one task, should take about 15 minutes. Based on the specified requirements, a storyboard and paper prototype were developed for the next phase.

### Development: Storyboard and paper prototyping (Phase 2.1)

In the development phase, we not only considered the requirements, but also made well thought-out choices regarding the game’s storyline to create a storyboard. In collaboration with the end users and our multidisciplinary team, the theme “space” was selected. Therefore, we decided to create tasks that were recognisable, but did not have a direct counterpart in everyday life. By adopting this approach, our aim was to enhance players’ ability to employ strategies in a task-independent manner, rather than solely improving their proficiency in the specific tasks during gameplay. In the game, players engaged in a search for “Dr. Karman,” a character portrayed as a famous scientist who went missing during a space expedition. The players’ mission was to ascertain the planet where Dr. Karman is presently situated. Throughout this challenging quest, players encountered a multitude of exciting adventures. The players began by planning their trip to the planet, assembling the necessary equipment and provisions, packing their bags, booking tickets for their space travel, and acquiring knowledge on how to harvest vegetables for sustenance. Upon arrival on the planet, they encountered several challenges like preparing meals with limited resources and launching a rocket. They even had the task of constructing their own rocket before embarking on the exciting space expedition. This adventurous space journey not only provided players a unique gaming experience but, more importantly, encouraged and stimulated them to utilize the GMT strategy during complex activities.

To enable players to acquire a strategy that is applicable in daily life, rather than just enhancing their proficiency in the game, we also diversified the ways in which the GMT strategy could be practiced across the different levels. For instance, it was decided to introduce individuals with the process of segmenting a goal into separate steps in a variety of ways: players had to arrange steps in the right order, generate steps based on a given route or set of rules, and distinguish relevant steps in a text with both relevant and irrelevant information (similar to situations encountered in daily life such as reading a recipe).

In the think-aloud sessions with end users, the primary focus of decision-making was centred around discussing the paper prototypes, aiming to enhance the user experience. Initial think-aloud sessions revealed that the games were overly complex and contained too many game elements. In order to narrow the focus and reduce cognitive load, it was decided to concentrate on one game element per game and on one single task that required players to employ the strategic approach. As a result of this simplification, end users rated the game as more transparent and manageable, which allowed them to focus more on honing their strategic abilities. In addition, we assessed the storyline and text. Initially, these contained an excessive amount of information, forming an additional burden for individuals with brain injuries. We chose to present the textual information in the game as speech bubbles to reduce cognitive load, limiting the amount of information to what was strictly required for game progression. In this respect, we omitted engaging and intriguing storylines, as well as background information about the significance of the GMT strategy and its explanations. We observed that the participants’ inability to recall these elements hindered their ability to perform the game. With this in mind, we began each game with a concise, informative text that outlined the importance of playing the game and practice the GMT strategy.

Taking the cognitive challenges of individuals with brain injuries into account, it was decided to grant players control over the game’s pace. This allowed people with brain injuries to process information at their own pace. Therefore, they could click an arrow to advance to the next screen or return to the previous screen to reread a sentence. By allowing this, complete comprehension and concentration on consolidating essential information by players was ensured.

Furthermore, with respect to the application (i.e., Plan Tool), it was decided to incorporate the functionality of image insertion, note-taking, and reminder setting to facilitate the execution of tasks or steps at designated times. Moreover, respondents with brain injury expressed the desire to resume a task following the completion of intervening tasks, as well as the opportunity to record accomplished goals within the application.

### Development: Iterative Developmental Process (Phase 2.2)

The main result from the iterative development process of the game could be summarized as follows: Ensuring a straightforward and simple game flow was considered the essential element. Moreover, it soon became apparent that providing guidance to players was crucial to minimize the energy spent on understanding gameplay and enable users to concentrate entirely on the application of the GMT strategy in various situations. To facilitate this, modifications were introduced to the game, including the utilization of an arrow to indicate the required interaction points, such as pointing out the button where users can find the step-by-step guide, the STOP button, and other key elements. Also, the game was designed with the intention of relieving players from the necessity of storing any information in memory. Instead, any potentially relevant data collected during gameplay was systematically stored within an easily accessible “information button,” allowing retrieval at any given moment.

**Figure 7:**
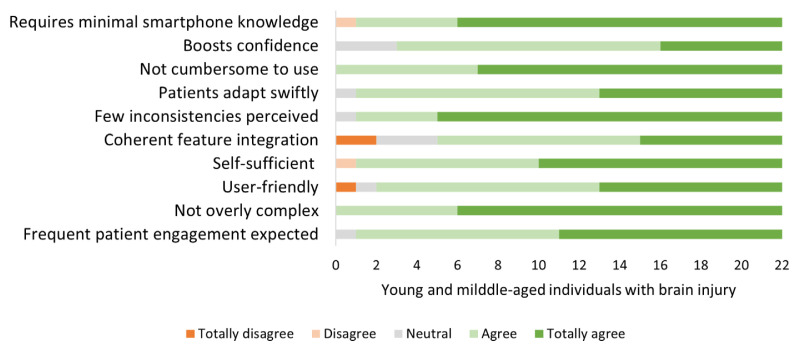
SUS Questionnaire Responses from Young and Middle-aged Individuals with Brain Injury (N = 22) at End of Development Phase 2 *Note*. Data provided insights into the Plan Tool’s usability.

Regarding the mobile application, simplification was also identified as an essential element. Through the feedback from individuals with brain injury gathered during the use of the application, we implemented necessary modifications to the app. These modifications were made to ensure that the individuals could operate the app independently, without requiring any additional explanation or information. A group of 22 individuals with brain injury, aged between 24 and 70 years, who experienced executive function complaints, assessed the usability of the Plan Tool using the SUS questionnaire [range: 0 – 100]. The results indicated a high level of usability, with an average score of 85.8 [range: 67.5 to 97.5]. The study participants expressed the desire to utilize the application on a regular basis, they exhibited a high level of self-assurance in their ability to operate it and required minimal instruction prior to its use. However, there was a greater variance in end-users’ perspectives concerning the cohesive integration of diverse features within the application, indicating that end-users had not yet reached a consensus on the extent to which the app’s features seamlessly and efficiently interact, provide a consistent user interface, synchronize data, and facilitate a user-friendly workflow (See Figure 7). This finding indicated the necessity of further prioritizing the optimization of the app’s functionality and the cohesive integration of its diverse features during development.

The data gathered from the think-aloud sessions, iterative testing sessions with end users, and the validated SUS questionnaire provided valuable insights that enabled us to pinpoint the particular areas that necessitated additional focus in the ongoing development process, to ensure successful implementation.

### Implementation in Rehabilitation Practice: Pilot Study (Phase 3.1)

After successfully integrating all suggestions gathered in the iterative development process into the games and mobile application, we proceeded with the addition of the Karman Line Plan module to the existing and abbreviated in-person GMT protocol. Although the assessment of usability was positive upon completion of the development process, it was evident that further improvements were necessary to ensure that the digital-supportive intervention could effectively contribute to therapy and be perceived as a valuable addition to therapy sessions by individuals with brain injury and therapists. The primary results of the implementation indicated a lack of adequate guidance within the game, which impeded individuals from achieving autonomous completion of the game levels without coming up against excessive frustration. Furthermore, a deficiency in the provision of corrective feedback for errors committed during gameplay was observed. Our analysis revealed that the feedback provided lacked sufficient specificity and required further elaboration in terms of literal and detailed formulation. It was necessary to achieve a balance between providing guidance during strategy application on the one hand, and the facilitation of autonomous execution of the strategy on the other hand.

**Figure 8:**
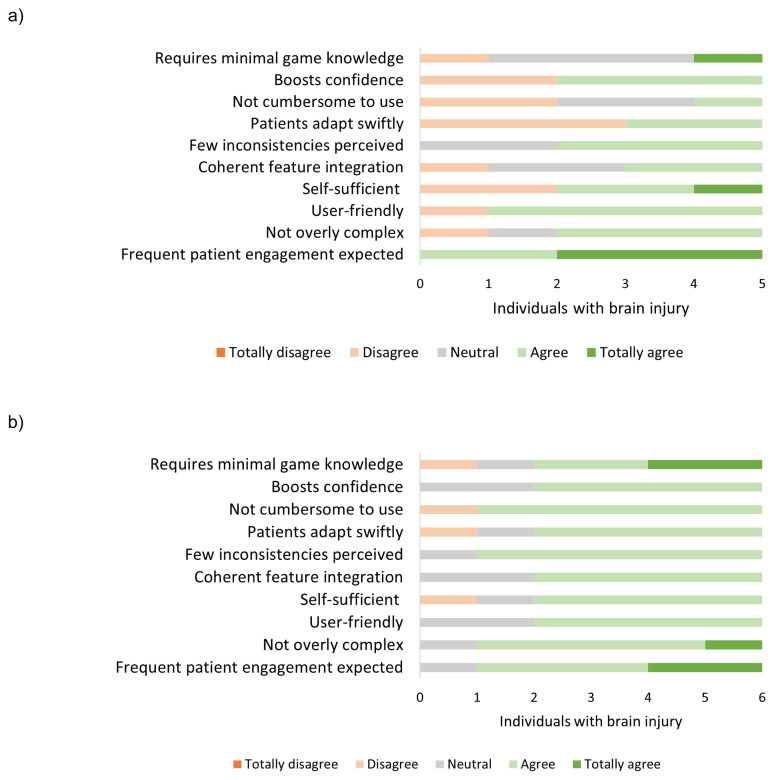
Plan Game: SUS Questionnaire Responses from Individuals with Brain Injury Who Were Referred to the Outpatient Rehabilitation Clinic During the Pilot Study, Providing insights into the Plan Game’s Usability. *Note*: Usability was measured at several time points, with lower usability scores observed primarily in the initial phase of the pilot study (8a, phase one). Higher scores were given later, after implementing the crucial features identified in the study (8b, phase two).

Usability was measured at several time points, with the resulting scores segmented into phase one and phase two. In the initial phase (phase one) of the pilot study, the usability scored by the end users with the SUS questionnaire varied between unacceptably low to good (*M* = 59.5; [range: 32.5 to 75]), see Figure 8a. Specifically, individuals with more executive function difficulties had greater difficulty and experienced more frustration while playing the game during this phase. As we introduced more structure and balance to the games (phase two) in an effort to provide more concise assistance, feedback, and encouragement in using the strategies, the usability scores became more consistent; fluctuating between acceptable and good (*M* = 69.6; [range: 55 to 80]), see Figure 8b. The same pattern was observed on the TAM questionnaire in terms of perceived enjoyment and perceived ease of use. Participants reported lower and variable scores on perceived enjoyment (*M* = 4.8; [range: 2.0 to 7.0]) and perceived ease of use (*M* = 4.7; range: [2.5 to 5.5]) at the start of the pilot study, as compared to the end of the study (perceived enjoyment (*M* = 5.5; [range: 4.0 to 7.0]) and perceived ease of use (*M* = 5.4; [range: 4.5 to 6.3])).

Importantly, throughout the pilot study, we did not make any crucial changes to the essence of the game or the instructional method for teaching the strategy. This consistency was evident in the perceived usefulness of the game, which remained consistent (phase one: *M* = 5.8; [range: 5.2 to 6.0]; phase two: *M* = 6.1; [range: 5.5 to 6.5]) throughout the entire pilot study. The participants reported that playing the game enhanced their ability to implement the strategy in their daily lives, particularly in terms of monitoring their behavior and facilitating the execution of daily tasks.

The participants’ attitudes toward using the game, as reflected by whether they would recommend it to other people with brain injury and/or their satisfaction with the game, increased similarly as did their perceptions of the game’s enjoyment and ease of use. Particularly in phase one, lower and more variable technology acceptance scores (*M* = 5.2; [range: 3.0 to 6.5]) were obtained; whereas in phase two, these scores had stabilized (*M* = 5.8; [range: 5.0 to 6.5]). Overall, participants rated the usability of the game as good, with a mean score of 65.00 [range: 32.5 to 80]. They enjoyed playing the game (*M* = 5.2, *SD* = 1.5; [range: 2.0 – 7.0]), had a positive attitude towards using the game in therapy (*M* = 5.5, *SD* = 1.05, [range: 3.0 – 6.5]), and indicated that the game was both easy to use (*M* = 5.0, *SD* = 1.1; [range: 2.5 – 3.6]) and useful (*M* = 6.0, *SD* = 0.43, [range: 5.2 – 6.5]), see Appendix A: Table A1.

**Figure 9:**
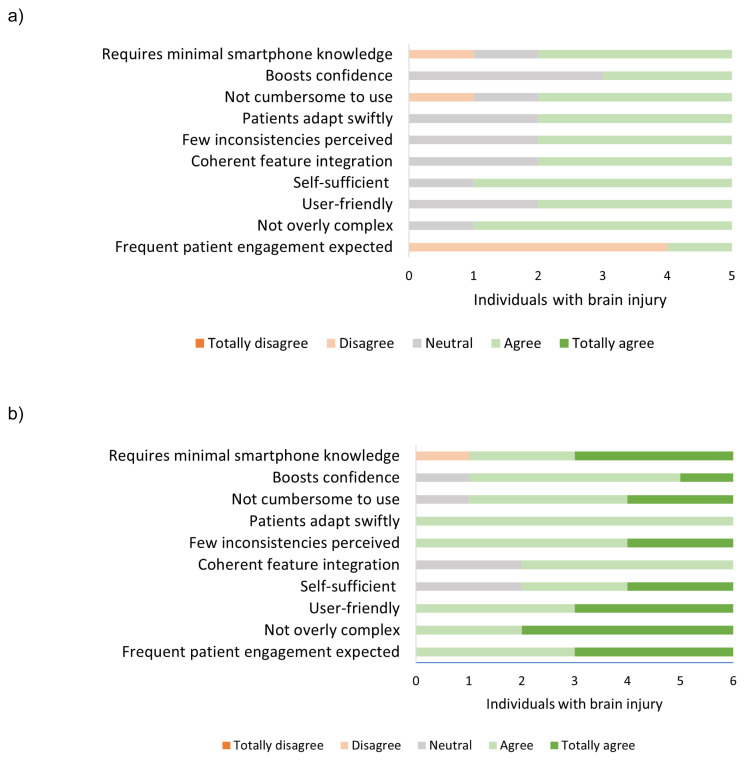
Plan Tool: SUS Questionnaire Responses from Individuals with Brain Injury from the Outpatient Clinic of Rehabilitation Centre Klimmendaal During the Pilot Study, Providing Insights into the Plan Tool’s Usability *Note*: Usability was measured during the pilot study, with lower usability scores observed in the initial phase of the pilot study (9a, phase one). Higher scores were achieved later, after implementing the features identified in the study (9b, phase two).

Upon implementing Plan Tool in rehabilitation practice (phase one), it became readily apparent that a number of elements were hindering usability. First, the feature of the phone’s screen having a limited time remaining active during task execution had not been taken into consideration. Second, the application required a greater number of input characters for entering activities. Lastly, the app required enhancements to make customizing steps simpler, such as allowing users to drag and drop steps to reorder them, adding the ability to insert new steps at any point in the sequence, and providing options to duplicate or delete specific steps. These limitations were also evident in the app’s usability scores, ranging from poor to good (*M* = 61.5; [range: 47.5 to 72.5], see Figure 9a) as measured by the SUS questionnaire, the level of enjoyment participants experienced while using the app (*M* = 4; range: [3.0 to 5.3]), the app’s perceived usefulness (*M* = 4.2; [range: 3.0 to 5.7]), and participants’ intent to continue using it (*M* = 3.9; [range: 2.5 to 6.0]). Despite these shortcomings, participants did express their willingness to utilize the app and deemed it usable, provided that the identified issues were resolved. After the resolution of these issues (phase two), participants’ evaluations of the mobile application’s usability improved, fluctuating between good and excellent (*M* = 80; [range: 75 to 87.5], see Figure 9b), they confirmed its utility (*M* = 6.1; [range: 5.0 to 6.7]) and expressed their intent to continue using it beyond the training period (*M* = 5.5; [range: 4.0 to 6.3]). Overall, participants rated the usability of the mobile application as good, with a mean score of 71.59 [range: 47.5 to 87.5], enjoyed using the app, had a positive attitude towards using the app, and indicated the app both easy to use and useful (see Appendix A: Table A2).

### Implementation in rehabilitation practice: Readiness approval by therapists (Phase 3.2)

The SUS questionnaires revealed that therapists rated the usability of both the games (*M* = 71.4 [range: 57.5 to 87.5]) and the app (*M* = 77.5 [range: 62.5 to 90]) as good. The TAM questionnaire revealed that therapists enjoyed using the game and the app, and found it easy to use during treatment. Moreover, almost all therapists recognized the high value and usefulness of the game and the app, expressing their intention to integrate them into the treatment (See Appendix A: Table A3 and Table A4). Therapists who rated the usefulness of either the game or the app as low were less willing to incorporate them into treatment.

## Discussion

### Principal Findings

The current study aimed to describe and evaluate the developmental process and usability of the Karman Line Plan module, a gamified Goal Management Training intervention for persons with brain injuries experiencing executive problems. A structured, user-centred approach was employed, encompassing three phases: (1) pre-development; (2) development prototype; and (3) implementation in rehabilitation practice.

In phase one, the pre-development phase, specific requirements and user characteristics were identified with input from the multidisciplinary team and individuals with brain injuries. It was decided to develop both a strategic game (i.e., Karman Line Plan Game) for tablet, laptop or PC use, and a mobile application (i.e., Karman Line Plan Tool) for smartphones. The Plan Game focuses on practicing the GMT strategy during pre-planned tasks and assignments, while the Plan Tool helps patients manage daily challenges, such as shopping or using public transport, by allowing them to create step-by-step plans and track task execution. Design choices, such as using soft colour tones and an isometric perspective, were also made based on user feedback.

In phase two, the development phase, the multidisciplinary team and patients made strategic decisions about the game’s storyline, created a paper prototype, and iteratively tested the digital prototype. The Plan Game was designed as an adventure point-and-click game where players search for Dr. Karman in space, performing tasks like planning a trip, packing a suitcase, and building a rocket. Sixteen tasks, each lasting 15–20 minutes, were developed to guide players through the GMT strategy. User feedback informed design adjustments were made, such as reducing information overload, allowing players to control the pace, and implementing silently moving visuals and structured feedback. For the GMT Tool, usability assessments by individuals with brain injuries indicated high overall usability. Think-aloud sessions and usability questionnaires with the endusers provided further insights for refinement.

Even so, when the Plan Tool was implemented in rehabilitation practice, a decline in perceived usability was noted in questionnaire ratings. Despite this, individuals with brain injuries consistently found both the Plan Game and Plan Tool useful, though they suggested modifications before clinical use. For the Plan Game, users needed more detailed instructions and specific feedback. For the Plan Tool, the modifications were more functional in nature, such as keeping the phone screen illuminated during tasks and increasing character limits for input. Rehabilitation centre’s broad staff of therapists (from occupational therapists to rehabilitation physicians) indicated the Plan Game and Plan Tool also as user-friendly, and as beneficial complements to their therapy sessions. Therapists linked the perceived usefulness to their intention to incorporate them into practice. To facilitate implementation, it therefore appears crucial to train therapists on how to integrate the digital treatment with in-person sessions and explain the development choices. In sum, the structured approach, combined with a multidisciplinary team of game developers, researchers, clinicians, and patients, collectively ensured the development of a user-friendly gamified compensatory strategy training for persons with brain injuries who experience executive problems.

### Limitations

Although the results of our study offer valuable insights, there are also some limitations that may have affected the outcomes and generalisability of the current study. First, one of the study’s limitations is the composition of the participant groups during the different phases of the developmental process. In the development phase (phase 2), the participant group included both young and middle-aged adults, while in the implementation phase (phase 3), only middle-aged adults participated. The difference in age groups might have affected the results, as there may be variations in cognitive ability and technological affinity between those age groups. However, because younger participants typically have more affinity with (the utilisation of) technical tools, this is not thought to have had a detrimental effect on usability scores in the implementation phase. Also, no differences in scores were found between young people and middle-aged individuals during phase 2 of development. Subsequently, our analysis did not take participants’ affinity for using technological tools into account, nor their motivation to use these tools in daily life. It is plausible that participants who volunteered to participate in the study in the development phase may have had more technological affinity than those who participated in the study as part of their rehabilitation trajectory in the implementation phase. However, in this case as well, it is possible that the differences in affinity and motivation just contributed to reduced scores during the implementation phase rather than an overestimation of the product’s usability.

Secondly, the study is limited by the fact that various methods were used to select participants during the development and implementation phases. In the development phase, participants were selected based on self-reported symptoms (i.e. without the use of questionnaires), while in the implementation phase, participants were included based on a validated self-report questionnaire (BRIEF-A) measuring subjective executive symptoms (i.e., complaints) in daily life. It is important to notice that some participants in the development phase may not have experienced the specific challenges related to executive function problems as participants included in the implementation phase. At the same time, this approach increased the generalisability of the study, as we were able to include a diverse group of participants with a wider range of experiences and backgrounds. Thirdly, the lack of further information on participants’ objective cognitive functioning, such as the results of neuropsychological tests, is another shortcoming. This information might have been relevant to investigate for which participants the digital contributions were best suited, especially given the large differences in (executive) functioning that were observed.

Given the wide range of executive function problems among people with brain injuries, tailoring the Plan module to the level of each individual appeared to be a difficult undertaking and was a significant challenge in the current study. In the future, AI algorithms could be utilised to better match the game levels to the individual’s abilities to address this constraint. Also, a clear performance report that highlights where individuals make errors in the game would be ideal for therapists to more easily incorporate this information into therapy sessions. In spite of these limitations, individuals with brain injuries and therapists rated the Plan Game levels as intuitive to play and viewed them as beneficial to their treatment. Future research should evaluate the effectiveness of the Karman Line Plan module for persons with brain injuries who experience executive function problems. Additionally, the Karman Line Plan module might be relevant for other patient populations with executive function impairments, such as individuals with Parkinson’s disease, ADHD, autism, or Korsakoff’s syndrome, where the GMT strategy is utilized as an evidence-based treatment. However, it is essential to also evaluate the Plan module’s usability, usefulness, and effectiveness within these different patient groups.

## Conclusions

To summarise, the current study showed that both individuals with brain injury and therapists working in a rehabilitation centre rated the Karman Line Plan module (encompassing the Plan Game and Plan Tool) as user-friendly and acknowledged its added value to clinical practice. Additionally, the study showed that it is crucial to involve the target patient population for whom digital training methods are created in the development process, beginning in an early stage with the elaboration of project plans/ideas, to make early adjustments and meet the demands and desires of the end user during the development process. The study also showed that frequent testing with the end user contributes to a user-friendly final product. This not only requires testing with the target patient population during the development phase but also requires monitoring the usability rated by participants and therapists when the module is implemented in clinical practice.

## Figures and Tables

**Figure 10:**
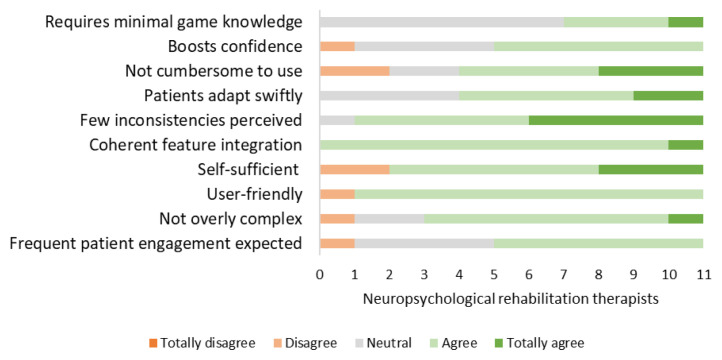
Plan Game: SUS Questionnaire Responses from Therapists Providing Insights into the Plan Game’s Usability

**Figure 11:**
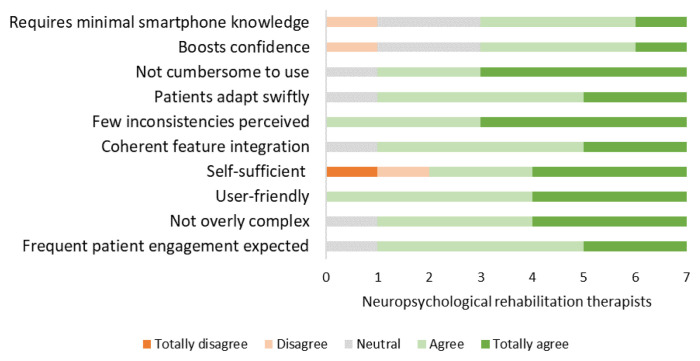
Plan Tool: SUS Questionnaire Responses from Therapists Providing Insights into the Plan Tool’s Usability

**Table 1:** Statements Used in the Survey to Assess the Level of Likelihood of Different Design Elements of the Plan Tool

Item	Measure
**1**	I think the lay-out of the app looks attractive
**2**	I found the colors used in the app not uncomfortable or unbearable
**3**	I think the information in the app is clearly organized
**4**	I think the text is presented in a logical position on the screen
**5**	I found the text in the app easy to read
**6**	I think the function of the buttons in the app are easy to understand
**7**	I found the buttons on the screen easy to operate
**8**	I think the character used in the app is attractive

*Note*. Participants rated the statements on a Likert scale from 1 (totally disagree) to 5 (totally agree)

**Table 2:** Characteristics of the Participating End Users in the Study

	Phase 1	Phase 2			Phase 3
	(*N* = 3)	Young (*N* = 10)	Middle-aged (*N* = 12)	Outpatient clinic (*N* = 1)	Outpatient clinic (*N* = 11)
**Age (mean ± SD)**	37.3 ± 15.6	27.4 ± 3.3	52.6 ± 6.3	67 ± 0	60 ± 7.4
**[min – max]**	[21–52]	[23–32]	[44–62]		[45–70]
**Women ** ***N*** ** (%)**	2 (67%)	7 (70%)	9 (75%)	0 (0%)	3 (27%)
**Types of Brain Injury ** ***N*** ** (%)**					
**ABI**	2 (67%)	2 (20%)	8 (67%)		6 (55%)
**TBI**	1 (33%)	6 (60%)	3 (25%)	1 (100%)	2 (18%)
**Encephalitis**		1 (10%)	2 (67%)		1 (0,1%)
**Tumour**		1 (10%)			2 (18%)
**Months post-injury (mean ± SD)**	48.7 ± 27.3	51.6 ± 47.8	45.3 ± 41.1	24 ± 0	22 ± 10.5
**[min – max]**	[26–79]	[6–168]	[12–144]		[6–36]

*Note*. Demographics are presented in *N* (%) unless stated otherwise. SD, standard deviation; ABI, acquired brain injury; TBI, traumatic brain injury.

## Abbreviations

GCT: gamified cognitive training
BRIEF-A: Behaviour Rating Inventory of Executive Function-Adult
CTT: Cognitive training therapists
GMT: Goal Management Training
NP: Neuropsychologists
OT: Occupational therapists
PC: Personal computer
SUS: System Usability Scale
TAM: Technology Acceptance Measure

## Appendix A

**Table A1:** Plan Game: Technology Acceptance Model (TAM) Questionnaire Rated by Individuals with Brain Injury (n = 11) in the Pilot Study

Variable	Questions	Mean* (SD); [min–max]
Perceived enjoyment	Q1: I enjoyed playing the game. Q2: I had fun while playing the game.	5.2 (1.5); [2.0 – 7.0]
Perceived ease of use	Q3: The interaction with the game was clear and understandable. Q4: Interacting with the game did not require much mental energy . Q5: I found the game easy to use. Q6: I found it easy to make the game do what I wanted it to do.	5.0 (1.1); [2.5 – 6.3]
Perceived usefulness	Q7: I found it useful to play the game. Q8: Playing the game helped me to apply the GMT strategy better during everyday activities. Q9: Playing the game made it easier to learn the GMT strategy myself. Q10: Playing the game helped me get a better overview when performing an everyday activity. Q11: Playing the game made me aware of the importance of checking myself after each step. Q12: Because I played the game, I can perform everyday activities better and easier than in the past.	6.0 (0.43); [5.2 – 6.5]
Intention to use	Q13: I will recommend other people with acquired brain injury to play the game. Q14: I am satisfied with the benefits I have gained from playing the game.	5.5 (1.05); [3.0 – 6.5]

* Ratings on a Likert scale with 1 corresponding to “completing disagree” and 7 corresponding to “completely agree”.

**Table A2:** Plan Tool: Technology Acceptance Model (TAM) Questionnaire Rated by Individuals with Brain Injury (n = 11) in the Pilot Study

Variable	Questions	Mean* (SD); [min–max]
Perceived enjoyment		4.8 (1.06); [3.0 – 6.3]
Perceived ease of use		5.5 (0.92); [3.0 – 6.6]
Perceived usefulness	Q1: The Tool is an enrichment in my daily life. Q2: I enjoy using the Tool. Q3: I had fun while using the Tool.	5.2 (1.25); [3.0 – 6.7]
Intention to use	Q4: Learning to use the Tool was easy for me. Q5: Interacting with the Tool was clear and understandable. Q6: Interacting with the Tool did not require much mental energy. Q7: I found the Tool easy to use. Q8: I found it easy to make the Tool do what I wanted it to do.	4.8 (1.36); [2.5 – 6.3]

* Ratings on a Likert scale with 1 corresponding to “completing disagree” and 7 corresponding to “completely agree”.

**Table A3:** Plan Game: Technology Acceptance Model (TAM) Questionnaire Rated by Therapists Working in the Rehabilitation Centre (n = 11) in the Pilot Study

Variable	Questions	Mean* (SD); [min–max]
Perceived enjoyment	Q1: I enjoyed playing the game. Q2: I had fun while playing the game.	5.8 (0.3); [5.0 – 6.0]
Perceived ease of use	Q3: The interaction with the game was clear and understandable. Q4: I don’t think using the game in treatment requires much mental energy. Q5: Learning to integrate/use the game in treatment seems easy to me. Q6: It seems easy to become skilled in using the game in treatment.	5.6 (0.4); [4.0 – 7.0]
Perceived usefulness	Q7: I think I would enjoy using the game in treatment. Q8: I find it useful to use the game in treatment. Q9: I think the game helps me to teach patients how to apply the strategy in everyday activities. Q10: By having patients play the game, I think it is easier for them to learn the strategy themselves. Q11: I think by playing the game, patients get a better overview while performing everyday activities. Q12: I think playing the game makes patients more aware of the importance of checking themselves after each step. Q13: I think that by playing the game, patients can perform everyday activities better and more easily than in the past. Q14: I think using the game increases the effectiveness of treatment.	5.5 (1.2); [3.0 – 7.0]
Intention to use	Q15: I will recommend other therapists to use the game in treatment. Q16: I am satisfied with the benefits we can get from using the game in treatment. Q17: Overall, I would find using the game in treatment useful. Q18: I plan to use the game regularly for cognitive rehabilitation in the near future. Q19: In the future, I prefer to use the game treatment rather than the traditional way for cognitive rehabilitation of patients.	5.3 (1.2); [3.0 – 7.0]

* Ratings on a Likert scale with 1 corresponding to “completing disagree” and 7 corresponding to “completely agree”.

**Table A4:** Plan Tool: Technology Acceptance Model (TAM) Questionnaire Rated by Therapists Working in the Rehabilitation Centre (n = 7) in the Pilot Study

Variable	Questions	Mean* (SD); [min–max]
Perceived enjoyment	Q1: I found the Tool enjoyable to use. Q2: I had fun while using the Tool.	6.3 (0.6); [5.0 – 7.0]
Perceived ease of use	Q3: The interaction with the Tool was clear and understandable. Q4: I don’t think using the Tool in treatment requires much mental energy. Q5: Learning to integrate/use the Tool in treatment seems easy to me. Q6: I think it would be easy to become proficient in using the Tool in treatment.	6.1 (0.6); [5.0 – 7.0]
Perceived usefulness	Q7: I think I would enjoy using the Tool in treatment. Q8: I find it useful to use the Tool in treatment. Q9: I think by using the Tool I can better teach patients how to apply the strategy during everyday activities. Q10: By having patients use the Tool, I think it is easier for them to learn the strategy themselves. Q11: I think by using the Tool, patients will get a better overview while performing everyday activities. Q12: I think by using the Tool, patients become more aware that it is important to check themselves after each step. Q13: I think that by using the Tool, patients are able to perform everyday activities better and more easily than in the past. Q14: I think by using the Tool the effectiveness of treatment will increase.	6.1 (0.6); [4.0 – 7.0]
Intention to use	Q15: I will recommend other therapists to use the Tool in treatment. Q16: I am satisfied with the benefits we can get from using the Tool in treatment. Q17: Overall, I would find using the Tool in treatment useful. Q18: I plan to use the Tool regularly for cognitive rehabilitation in the near future. Q19: In the future, I prefer to use the treatment in combination with the Tool rather than the traditional way for cognitive rehabilitation of patients.	6.1 (0.8); [4.0 – 7.0]

* Ratings on a Likert scale with 1 corresponding to “completing disagree” and 7 corresponding to “completely agree”.
